# Psychogenic nonepileptic seizures in adult neurology clinics in southern Iran: A survey of neurologists

**Published:** 2016-04-03

**Authors:** Ali Asadi-Pooya

**Affiliations:** ^1^ Neuroscience Research Center, School of Medicine, Shiraz University of Medical Sciences, ‎Shiraz, Iran AND Jefferson Comprehensive Epilepsy Center, Department of Neurology, Thomas ‎Jefferson University, Philadelphia, USA

**Keywords:** Seizures, Epilepsy, Psychogenic, Diagnosis, Perception, Practice, Iran

## Abstract

**Background:** We investigated the perceptions of the neurologists practicing in Fars province in Southern Iran about psychogenic nonepileptic seizures (PNES); their diagnostic processes and management strategies.

**Methods:** In this survey, all neurologists participating at the annual meeting of neurologists were asked to participate. These neurologists practice in Fars province. An anonymous questionnaire was specifically developed for this study.

**Results:** About 18 neurologists (14 males and four females), out of 20 attendees, agreed to participate in the study. The mean age of the participants was 41.6 ± 7.5 years. They estimated that 10.8% of patients attending their clinic had seizures or blackouts, whereas 4.4% of patients attending their clinic had PNES. The experiences of the participants about the manifestations that potentially differentiate PNES from epileptic seizures; the tests they use to diagnose suspected patients and their treatment strategies showed significant variability. For example, the tests the neurologists always used for the diagnosis of PNES in suspected patients included routine electroencephalographs (EEGs) by 9 (50%), video-EEG monitoring by 4 (22%), and serum creatine phosphokinase (CPK) measurement by 2 (11%).

**Conclusion:** There is much variability in the approaches to diagnosis and management of PNES in southern Iran. The participants in our study were aware of the many knowledge gaps in this area.

## Introduction

Psychogenic nonepileptic seizures (PNES) are relatively common reason why patients attend epilepsy clinics.^1^^-^^5^ In patients with PNES, it takes a mean of more than 5 years to reach to a correct diagnosis and most of these patients receive inappropriate treatment with antiepileptic drugs (AEDs).^1^ This observation demonstrates that most physicians continue to struggle with the correct diagnosis of PNES and their distinction from epileptic seizures. Patients with PNES are at risk of iatrogenic harm, as they are more likely to receive inappropriate medications, hospital admissions, and emergency treatments.^6^^,^^7^

In this study, we investigated the perceptions of the neurologists practicing in Fars province in Southern Iran about PNES, the diagnostic processes and management strategies for this disorder, to identify possible education and training needs.

## Materials and Methods

In this survey, all 20 neurologists present at the annual meeting of the neurologists were asked to participate. These neurologists practice in Fars province. An anonymous questionnaire was developed for this study. This questionnaire included questions about the participant’s gender, age, years in practice, place of practice, and also questions with regard to the epidemiology of PNES at their clinics, manifestations and tests that potentially differentiate PNES from epileptic seizures in suspected patients, and their treatment strategy in patients with PNES. Their answers were summarized descriptively and analyzed anonymously. This study was conducted with the approval by Shiraz University of Medical Sciences Review Board.

## Results

A total of 18 neurologists (14 males and 4 females) agreed to participate. All respondents completed over 90% of the individual items on the questionnaire. The mean age [± Standard deviation (SD)] of the participants was 41.6 ± 7.5 years. They were in practice for 8.9 ± 7.9 years (range: 1-30 years). Four participants were in academic practice, seven neurologists were in private, and seven others were both in academic and private practice.

They estimated that 10.8 ± 15.3% (range: 1-70%) of patients attending their clinic had seizures or blackouts, whereas 4.4 ± 6.8% (range: 0.1-30%) of patients attending their clinic had PNES. Their estimate was that 7.0 ± 9.5% (range 0-30%) of patients with PNES attending their clinic had both epilepsy and PNES. Finally, they said that 77.2 ± 15.2% (range: 40-90%) of patients with PNES attending their clinic were women. 

The experiences of the participants about the manifestations that potentially differentiate PNES from epileptic seizures are shown in table 1. 

**Table 1:** The experiences of the participants about the manifestations that potentially differentiate psychogenic nonepileptic seizures (PNES) from epileptic seizures*

**Clinical manifestation**	**Only in epilepsy**			**Mostly in epilepsy**	**Equally common in epilepsy and PNES**	**Mostly in PNES**	**Only in PNES**
Generalized fine shaking (tremor)	0			1	3	11	1
Generalized violent shaking	0			2	9	7	0
Focal shaking (in one limb or one side of the body)		0		8	5	5	0
Altered consciousness	0			9	9	0	0
Asynchronous limb movements	0			1	3	14	0
Out of phase clonic activity	0			3	1	12	0
Intermittent or waxing and waning motor activity			0	0	3	15	0
Pelvic movements (forward thrusting)	0			0	0	13	5
Side to side head movement	0			0	0	14	4
Eyes closed during convulsive seizure	0			0	6	11	1
Resisted eyelid opening	0			0	0	14	4
Dystonic limb movements and opisthotonus back arching	0			5	2	11	0
Gradual onset and cessation of seizures	1			2	4	11	0
Ictal crying, weeping	0			6	2	8	2
Postictal crying, weeping	0			0	5	12	1
Prolonged seizures (more than 2-3 minutes)	0			0	4	13	1
Emotional or situational trigger for the seizures	0			0	3	14	0
Seizures provoked by suggestion	0			0	1	10	6
Aura	5			11	1	1	0
Urinary incontinence	2			15	1	0	0
Fecal incontinence	4			14	0	0	0
Nocturnal seizures	5			11	2	0	0
Ictal injury	3			13	0	0	0
Seizures lasting more than 5 minutes	0			1	3	13	1
High seizure frequency (several per week)	0			0	8	10	0
Clustering of seizures	0			0	7	10	0
No response to AEDs	0			0	3	15	0

AED: Antiepileptic drug; PNES: Psychogenic nonepileptic seizures

* Some answers were missing.

The tests the neurologists always used for the diagnosis of PNES in suspected patients included routine electroencephalographs (EEGs) by 9 (50%), video-EEG monitoring by 4 (22%), and serum creatine phosphokinase (CPK) measurement by 2 (11%). Only 5 (28%) neurologists said they always discontinue the AEDs and 12 (67%) said they always refer the patient to a psychologist or psychiatrist. 10 (56%) neurologists said that they tended to follow the patients up until AEDs are withdrawn, and 4 (22%) followed the patients up until seizures were controlled. 11 neurologists (61%) believed that it is very helpful and five persons (28%) said that is somewhat helpful to attend a teaching course or symposium about different aspects of PNES to improve their practice.

## Discussion

In this survey, we investigated the perception and the clinical approach of the participating neurologists to PNES in Southern Iran. The respondents’ estimate of the patients with PNES attending their clinics (4.4%) showed that PNES are relatively common even in general neurology clinics. This observation has been repeatedly mentioned in previous studies.^1^^,^^8^

The participants in our study thought that about 7.0% of patients with PNES attending their clinic had both epilepsy and PNES. This figure is different from what we observed in our previous study in the same region when the patients were investigated thoroughly with prolonged video-EEG recordings (17.0%).^9^ This difference probably reflects the challenges the neurologists face in making a correct diagnosis in suspected patients.^10^ This challenge was clearly highlighted when we asked about the experiences of the participants about the manifestations that potentially differentiate PNES from epileptic seizures (Table 1). In addition, we observed that there was confusion among the neurologists in our region with respect to the tests used for the diagnosis in patients suspected of having PNES. A similar observation has previously been reported from the UK.^8^

More frequent use of video-EEG monitoring may allow neurologists to make a definitive diagnosis more often. This will reduce the inappropriate use of AEDs and direct patients with PNES to more appropriate forms of treatment. A definitive diagnosis also reduces the risk of over diagnosing PNES in patients with epilepsy or emotional problems.^8^ When asked about their treatment and follow-up strategies, the variability of approaches among the neurologists was as great as that variability in their diagnostic processes. Again, this observation has been reported in previous studies.^8^

## Conclusion

The findings of our study show that there is much variability in the approaches to diagnosis and management of PNES. The participants in our study were aware of the many knowledge gaps in this area: About 90% of the respondents endorsed the need to attend a teaching course or symposium about different aspects of PNES to improve their practice.

## Notes:

**How to cite this article:** Asadi-Pooya A. Psychogenic nonepileptic seizures in adult neurology clinics in southern Iran: A survey of neurologists. Iran J Neurol 2016; 15(2): 100-2.
